# Bayesian statistics in the design and analysis of cluster randomised controlled trials and their reporting quality: a methodological systematic review

**DOI:** 10.1186/s13643-021-01637-1

**Published:** 2021-03-31

**Authors:** Benjamin G. Jones, Adam J. Streeter, Amy Baker, Rana Moyeed, Siobhan Creanor

**Affiliations:** 1 Medical Statistics, Faculty of Health: Medicine, Dentistry and Human Sciences, University of Plymouth, Room N15, ITTC Building 1, Plymouth Science Park, Plymouth, Devon PL6 8BX UK; 2 NIHR ARC South West Peninsula (PenARC), College of Medicine and Health, University of Exeter, Exeter, Devon UK; 3 Klinische Epidemiologie, Institut für Epidemiologie und Sozialmedizin, Westfälische Wilhelms-Universität Münster, Münster, Germany; 4 School of Computing, Electronics and Mathematics, Faculty of Science and Engineering, University of Plymouth, Plymouth, Devon UK; 5 Peninsula Clinical Trials Unit, Faculty of Health: Medicine, Dentistry and Human Sciences, University of Plymouth, Plymouth, Devon UK; 6 Exeter Clinical Trials Unit, College of Medicine and Health, University of Exeter, Exeter, Devon UK

**Keywords:** Cluster randomised trial, Bayesian, CONSORT statement, Sample size, Statistical power, Hierarchical modelling

## Abstract

**Background:**

In a cluster randomised controlled trial (CRCT), randomisation units are “clusters” such as schools or GP practices. This has methodological implications for study design and statistical analysis, since clustering often leads to correlation between observations which, if not accounted for, can lead to spurious conclusions of efficacy/effectiveness. Bayesian methodology offers a flexible, intuitive framework to deal with such issues, but its use within CRCT design and analysis appears limited. This review aims to explore and quantify the use of Bayesian methodology in the design and analysis of CRCTs, and appraise the quality of reporting against CONSORT guidelines.

**Methods:**

We sought to identify all reported/published CRCTs that incorporated Bayesian methodology and papers reporting development of new Bayesian methodology in this context, without restriction on publication date or location. We searched Medline and Embase and the Cochrane Central Register of Controlled Trials (CENTRAL). Reporting quality metrics according to the CONSORT extension for CRCTs were collected, as well as demographic data, type and nature of Bayesian methodology used, journal endorsement of CONSORT guidelines, and statistician involvement.

**Results:**

Twenty-seven publications were included, six from an additional hand search. Eleven (40.7%) were reports of CRCT results: seven (25.9%) were primary results papers and four (14.8%) reported secondary results. Thirteen papers (48.1%) reported Bayesian methodological developments, the remaining three (11.1%) compared different methods. Four (57.1%) of the primary results papers described the method of sample size calculation; none clearly accounted for clustering. Six (85.7%) clearly accounted for clustering in the analysis. All results papers reported use of Bayesian methods in the analysis but none in the design or sample size calculation.

**Conclusions:**

The popularity of the CRCT design has increased rapidly in the last twenty years but this has not been mirrored by an uptake of Bayesian methodology in this context. Of studies using Bayesian methodology, there were some differences in reporting quality compared to CRCTs in general, but this study provided insufficient data to draw firm conclusions. There is an opportunity to further develop Bayesian methodology for the design and analysis of CRCTs in order to expand the accessibility, availability, and, ultimately, use of this approach.

## Background

In a cluster randomised controlled trial (CRCT), randomisation occurs at the group (or “cluster”) level as opposed to the individual level that is typical in traditional Randomised Controlled Trials (RCTs). Examples of naturally-occurring clusters include schools, villages and GP practices. Randomisation of clusters, rather than individuals, is conducted for a number of reasons: (i) when the intervention is to be delivered at the cluster level (e.g. to a whole school/class within a school); (ii) when there is a risk of contamination, either between participants or those delivering the intervention; or (iii) when there is a clear administrative, logistic or cost-based rationale [1].

Cluster randomisation has methodological implications that go beyond merely the randomisation procedure itself. Measurements on individuals within the same cluster are likely to be more correlated to one another than measurements on individuals from different clusters. This correlation creates an additional level of complexity, which must be accounted for in both the study design and sample size calculation, and the statistical analysis. Failure to do so can result in an underpowered study and ultimately spurious conclusions about the efficacy or effectiveness of the intervention or treatment under investigation.

**Fig. 1:**
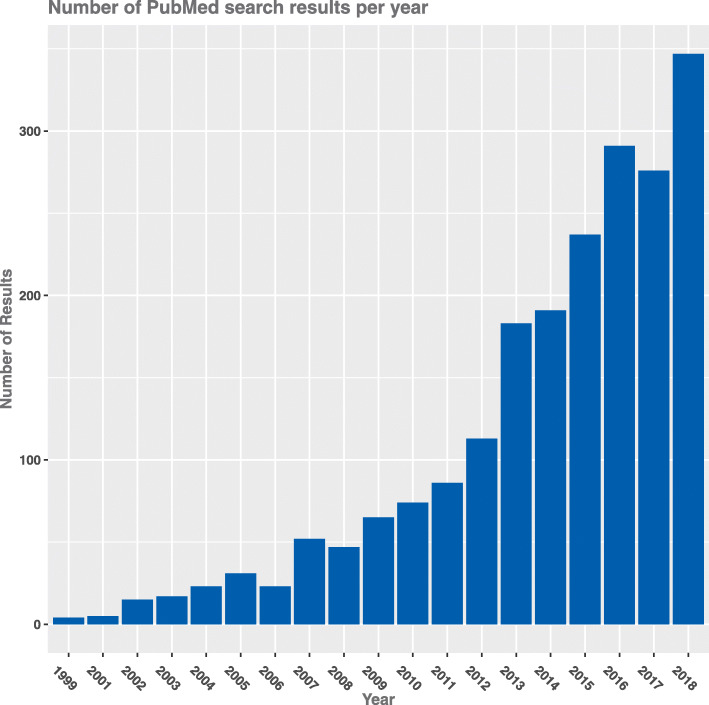
Number of PubMed search results per year. Search term: “cluster randomized controlled trial”[Title] OR “cluster randomised controlled trial”[Title] NOT “stepped”[Title]. The search was conducted in February 2019 and partial data for that year was removed

CRCTs are a relatively novel study design, but the methodology is now well established in the literature. Prior to the 1980s, there was only sparse use of CRCTs [2], but they have become increasingly popular in the last 30 years, from just seven reported in 1990, to over 120 in 2008 [3, 4]. Figure 1 provides an illustration of this increase in popularity by displaying the number of search results by year for “cluster randomised controlled trials” with restriction to publication title. Alongside such a rapid increase in the use of the CRCT design, there have been some attempts to develop new Bayesian methodology for the design and analysis of such trials. This ranges from utilising well-established Bayesian hierarchical modelling approaches to account for the clustered nature of the data [5], through to more novel approaches to study design and sample size calculation such as that developed by Turner et al [6, 7]. The Bayesian approach to analysis in particular may offer a number of advantages over the frequentist approach. In a random effects setting, as is often applicable in the analysis of a CRCT, the hierarchical Bayesian framework provides a flexible, intuitive approach to statistical inference. Furthermore, Bayesian analysis facilitates a more natural, probabilistic interpretation of results and moves away from frequentist hypothesis testing and *p*-values, an approach which has been criticised in recent years [8]. Whilst often criticised, the incorporation of prior information into a statistical analysis can facilitate more informative conclusions, which reflect all the available evidence as opposed to simply the evidence offered from the single dataset at hand. In many cases, the rationale for the inclusion of informative priors is sound, for example results from previous research or even existing data (such as pilot or feasibility studies). However, whilst the advantages of the Bayesian approach to both the analysis of clinical trials [9] and hierarchical data [10] are clear and have been documented, it is unclear whether such methods are being regularly utilised within the context of CRCTs.

With the increased use of CRCTs, the need for consistent, high-quality reporting is crucial. In response to this recognised need, the CONSORT extension to cluster randomised trials was first published in 2004 [11] and updated in 2012 [12]. The CONSORT statement provides recommendations for reporting of randomised trials, and whilst there is no extension for Bayesian trials, it was not written exclusively for frequentist methods. A recent review of the methodological quality of sample size calculations in a sample of 300 CRCTs published between 2000 and 2008, found that only 55.3% (166) presented a sample size calculation, of which only 61.4% (102) accounted appropriately for clustering [13]. A separate recently published review of the same sample of CRCTs examined the impact of the 2004 CONSORT extension on more general methodological quality and concluded that adherence to published reporting guidelines and quality remains low [14]. Similar reviews of CRCT reporting quality have been conducted and produced comparable conclusions [15, 16]. However, to our knowledge, none have focussed specifically on CRCTs which incorporated Bayesian methods, and so both the quantity and quality of these are unknown.

This review aims to:

(i) Quantify and explore the use of Bayesian methodology in the design and/or analysis of CRCTs;

(ii) Appraise the quality of reporting of CRCTs conducted in a Bayesian framework against the current relevant CONSORT guidelines and identify whether the reporting quality differs from previous reviews assessing reporting quality in CRCTs more generally (most of which likely, but not necessarily, pertain to frequentist trials).

The impact of the introduction of the CONSORT guidelines for CRCTs in 2004 and 2012 on reporting quality will also be appraised.

## Methods

The protocol for this methodological systematic review was developed prospectively and made publically available online [17] before commencing the literature searches. The review was conducted and reported in accordance with the PRISMA guidelines [18].

### Inclusion and exclusion criteria

We sought to identify all published parallel group CRCTs in which Bayesian methodology was used in either the study design (including sample size calculation) or statistical analysis. We also opted to include any papers in which Bayesian methodology was discussed or considered, even if such methods were not implemented in the study, whilst recognising that such a scenario would be unlikely. We did not restrict our search or inclusion on the basis of publication date, location, intervention type or population in any way, provided the relevant paper was published in the English language, due to resource limitations.

In order to be included in this review, it had to be evident that randomisation in the study occurred at a group level, in which multiple participants were randomised together, as per the definition of a CRCT.

We did not exclude references on the basis of type (category) of published paper. Specifically, we included not only primary reports of efficacy or effectiveness but also protocol papers, papers reporting secondary analyses and publications reporting results of pilot/feasibility studies. We also included studies reporting Bayesian methodological developments in the area of CRCTs. At the data extraction stage, we sought to identify supplementary literature related to the same study, if indicated, to obtain the required information, but only included such examples as a single entry. It was anticipated, for example, that this might include obtaining additional detail from a published protocol or monograph that had been omitted in the corresponding primary results paper.

We excluded papers reporting only cost-effectiveness. We also excluded studies implementing a stepped-wedge or other longitudinal cluster randomised design, as the methodological considerations are different and the reporting quality metrics presented in the CONSORT extension to CRCTs [12] are not valid for such longitudinal designs. Since commencement of this systematic review, however, separate guidelines for stepped-wedge designs have been published [19]. Conference proceedings and masters and PhD dissertations were not included.

### Data sources and search methods

We searched both Medline and Embase using Ovid, as well as the Cochrane Central Register of Controlled Trials (CENTRAL), for relevant publications on 24 July 2018, without restriction on date of publication. The full electronic search strategy was an extension of that presented by Taljaard et al. [20] to identify CRCTs, adapted to identify only studies including the word “Bayes” in the title, abstract or text. The full electronic search strategy used to search Medline and Embase is shown in Table 1, with minor syntactic adaptations required in order to run the search in CENTRAL. The searches were undertaken by BJ. Additional literature was included where appropriate through hand searching of the authors’ own collection of references.

**Table 1:** Search strategy used to search Medline and Embase within Ovid on 24 July 2018

#	Search
**Existing published strategy for randomised controlled trials**	
1	(article OR randomized controlled trials).pt.
2	Animals/
3	Humans/
4	#2 NOT (2 AND 3)
5	#1 NOT #4
**Cluster design–related terms**	
6	(cluster$ adj2 randomi$).tw.
7	((communit$ adj2 intervention$) or (communit$ adj2 randomi$)).tw.
8	group$ randomi$.tw.
9	#6 OR #7 OR #8
10	intervention?.tw.
11	Cluster Analysis/
12	Health Promotion/
13	Program Evaluation/
14	Health Education/
15	#10 OR #11 OR #12 OR #13 OR #14
16	#9 OR #15
**Bayesian search terms**	
17	bayes$.af.
18	#16 AND #17
**Final search**	
19	#18 AND #5
20	limit #19 to (randomized controlled trial)

pt. represents publication type; / represents MeSH search; $ allows for truncation of words; adj allows for adjacency between search words; tw represents text words in abstract and/or title; af represents all fields; ? is a wildcard which retrieves one or 0 characters

### Reference sifting and quality control

After conducting electronic searches, all references were downloaded and imported to Mendeley [21] for electronic deduplication. Following this, remaining references were exported and uploaded to Rayyan [22]. BJ and AS independently reviewed each reference and made a decision to include or exclude on the basis of the information available from the title and the abstract assessed against the pre-specified inclusion/exclusion criteria outlined in the protocol [17]. Rayyan includes a blinding feature, which was switched on during the independent sifts and then disabled. Any disagreements were resolved through discussion and, where required, SC made a final decision.

After the initial sift, full-text articles were obtained for all remaining references. BJ examined the full texts and again made inclusion/exclusion decisions using Rayyan. SC or AB re-examined approximately half each of all full texts and independently made inclusion or exclusion decisions. Any disagreements were once again resolved through further discussion.

### Data extraction

For the primary and secondary published reports of trial results, we collected a range of data including demographic data, technical detail regarding design and analysis methodology with relation to Bayesian techniques, and information regarding statistician involvement with the study and their respective affiliations. For papers reporting primary results, we also collected a selection of reporting quality metrics taken from the 2012 CONSORT extension to CRCTs [12]. In addition, we recorded whether or not *p*-values were reported for comparison of baseline demographics, as has been collected in previous systematic reviews of CRCTs [15, 23], Clinical Trial Unit (CTU) involvement in the study, and journal endorsement of the CONSORT guidelines.

We considered the paper as having statistician involvement, via a previously used criterion [15, 24, 25], if there was a clearly designated statistician, or if at least one of the co-authors belonged to a department of epidemiology or biostatistics. If it was not possible to obtain this information from the authorship list on the paper, online searching was undertaken to attempt to determine this from the qualification or affiliation of the authors. In any cases where it was not possible to obtain the required information, statistician involvement was recorded as “no”. We also recorded the statistician’s affiliation to a CTU, an academic statistical department, a commercial pharmaceutical company, a clinical research organisation (CRO) or “other”. CTU involvement in the study was determined if at least one author had a listed affiliation to a CTU. If author affiliations were not available in the paper or online, this was recorded as “no”.

We classified journal endorsement of the CONSORT statement using previously defined criteria [15]: a journal’s strength of endorsement was classified as high if the words “required”, ”must”, ”should” or “strongly recommended” were used in their author instructions, a medium endorser if words “encouraged”, ”recommended”, “advised” or “please” were used, and a low endorser if “may wish to consider” or ”see CONSORT” was used. We included a fourth category, “none”, if the journal included no mention of the CONSORT statement in its guidelines to authors.

Separate data extraction forms were developed for primary and secondary results papers to ensure that all the required information was obtained independently, consistently and without bias. The forms were piloted by BJ prior to data extraction. Formal data extraction was not undertaken for the methodological papers, but rather these papers were examined for the purpose of qualitative reporting and descriptive summaries of the methods developed in order to gain an understanding of the extent of methodological developments in this area.

BJ conducted data extraction on all primary and secondary results papers. SC, AB and AS independently conducted approximately one-third each of the data extraction on all papers, and final data was agreed by the whole study team. BJ and SC also each independently classified the results papers as primary or secondary. Any disagreements were resolved through discussion. Separately, BJ examined the methodological papers for qualitative reporting, but no second data extraction was undertaken. BJ double-entered all data from the data extraction forms into separate excel spreadsheets for primary and secondary papers.

### Analysis

We present descriptive statistics of frequencies and percentages or means and standard deviations, as appropriate, for demographic qualities relating to each of the results publications, including trial location, number of participants recruited and type of primary outcome, by category of published results (primary or secondary). For the reporting quality measures, we present the number of primary results papers satisfying each criterion overall, by year (before or after the publication of the 2012 extension to the CONSORT guidelines for CRCTs [12]), by journal endorsement of the CONSORT guidelines (high or medium versus low or none) and by statistician involvement in the trial. We also summarise the use or consideration of Bayesian methods in the design and/or sample size calculation and/or analysis, as well as the level of information incorporated into the prior distributions specified. We also outline for which parameters the prior distributions were specified, if this information was available. Finally, a qualitative synthesis of the methodological papers was undertaken to summarise the areas of focus in the development of new methods.

## Results

**Fig. 2:**
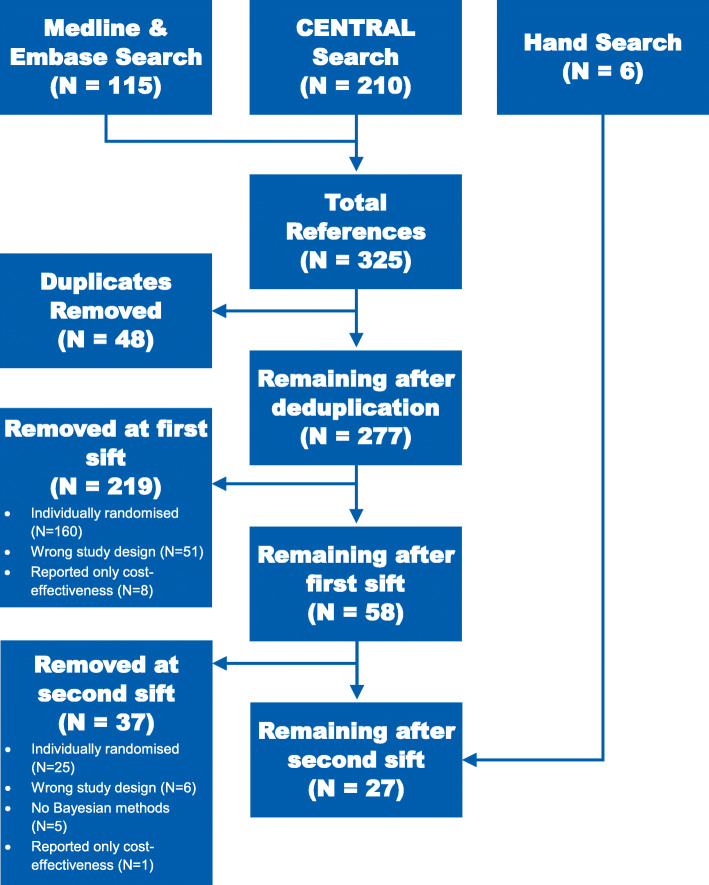
Flow diagram of the identification process for the 27 publications included in this review

We identified 325 records from our electronic searches, of which 48 were identified as duplicates and removed. The remaining 277 records were screened on the basis of the detail available within the title and abstract, of which 219 were excluded (51 were the wrong study design (such as *N*-of-1 trials or meta analyses), 160 were individually randomised trials, and eight were papers reporting cost-effectiveness only). Full texts were obtained for the remaining 58 papers. At this final stage, following independent review of the full texts, a further 37 were removed (25 were individually randomised, five did not include any mention of Bayesian methodology, six were the wrong study design and one paper reported only cost-effectiveness results), leaving 21 papers from the electronic search. A further six papers, all of which were methodological, were added through additional hand searches, resulting in a total of 27 papers included (Fig. 2). The full list of references for the included papers is detailed in Table 2. Eleven (40.7%) were reports of CRCT results, of which seven (63.6%, R1–R7) were primary results papers and four (36.4%, R8–R11) reported secondary analyses. Thirteen papers (48.1%, M1–M13) reported methodological developments and the remaining three (11.1%, C1–C3) reported comparisons of methods, assessing the performance of various existing methodology.

**Table 2:** References included in the review

**R1**	Carabin H, Millogo A, Ngowi HA, et al. Effectiveness of a community-based educational programme in reducing the cumulative incidence and prevalence of human Taenia solium cysticercosis in Burkina Faso in 2011–14 (EFECAB): a cluster-randomised controlled trial. *Lancet Glob Heal*. 2018;6(4):e411-e425. doi:10.1016/S2214-109X(18)30027-5
**R2**	Foxcroft DR, Callen H, Davies EL, Okulicz-Kozaryn K. Effectiveness of the strengthening families programme 10-14 in Poland: Cluster randomized controlled trial. *Eur J Public Health*. 2017;27(3):494-500. doi:10.1093/eurpub/ckw195
**R3**	Levy BT, Hartz A, Woodworth G, Xu Y, Sinift S. Interventions to Improving Osteoporosis Screening: An Iowa Research Network (IRENE) Study. *J Am Board Fam Med*. 2009;22(4):360-367. doi:10.3122/jabfm.2009.04.080071
**R4**	Ngowi HA, Carabin H, Kassuku AA, Mlozi MRS, Mlangwa JED, Willingham AL. A health-education intervention trial to reduce porcine cysticercosis in Mbulu District, Tanzania. *Prev Vet Med*. 2008;85(1-2):52-67. doi:10.1016/j.prevetmed.2007.12.014
**R5**	Rahme E, Choquette D, Beaulieu M, et al. Impact of a general practitioner educational intervention on osteoarthritis treatment in an elderly population. *Am J Med*. 2005;118(11):1262-1270. doi:10.1016/j.amjmed.2005.03.026
**R6**	Swanson KM, Chen H-T, Graham JC, Wojnar DM, Petras A. Resolution of Depression and Grief during the First Year after Miscarriage: A Randomized Controlled Clinical Trial of Couples-Focused Interventions. *J Women’s Heal*. 2009;18(8):1245-1257. doi:10.1089/jwh.2008.1202
**R7**	Van Deurssen E, Meijster T, Oude Hengel KM, et al. Effectiveness of a Multidimensional Randomized Control Intervention to Reduce Quartz Exposure among Construction Workers. *Ann Occup Hyg*. 2015;59(8):959-971. doi:10.1093/annhyg/mev037
**R8**	Amza A, Kadri B, Nassirou B, et al. Community risk factors for ocular chlamydia infection in Niger: Pre-treatment results from a cluster-randomized trachoma trial. *PLoS Negl Trop Dis*. 2012;6(4). doi:10.1371/journal.pntd.0001586
**R9**	Hovi T, Ollgren J, Savolainen-Kopra C, T. H, J. O. Intensified hand-hygiene campaign including soap-and-water wash may prevent acute infections in office workers, as shown by a recognized-exposure -adjusted analysis of a randomized trial. *BMC Infect Dis*. 2017;17(1):47. doi:10.1186/s12879-016-2157-z
**R10**	Barlis P, Regar E, Serruys PW, et al. An optical coherence tomography study of a biodegradable vs. durable polymer-coated limus-eluting stent: A LEADERS trial sub-study. *Eur Heart J*. 2010;31(2):165-176. doi:10.1093/eurheartj/ehp480
**R11**	See CW, O’Brien KS, Keenan JD, et al. The effect of mass azithromycin distribution on childhood mortality: Beliefs and estimates of efficacy. *Am J Trop Med Hyg*. 2015;93(5):1106-1109. doi:10.1111/sjos.12316
**M1**	Alexander N, Emerson P. Analysis of incidence rates in cluster-randomized trials of interventions against recurrent infections, with an application to trachoma. *Stat Med*. 2005;24(17):2637-2647. doi:10.1002/sim.2138
**M2**	Clark AB, Bachmann MO. Bayesian methods of analysis for cluster randomized trials with count outcome data. *Stat Med*. 2010;29(2):199-209. doi:10.1002/sim.3747
**M3**	Nixon RM, Duffy SW, Fender GR. Imputation of a true endpoint from a surrogate: Application to a cluster randomized controlled trial with partial information on the true endpoint. *BMC Med Res Methodol*. 2003;3:1-11. doi:10.1186/1471-2288-3-17
**M4**	Olsen MK, DeLong ER, Oddone EZ, Bosworth HB. Strategies for analyzing multilevel cluster-randomized studies with binary outcomes collected at varying intervals of time. *Stat Med*. 2008;27(29):6055-6071. doi:10.1002/sim.3446
**M5**	Thompson SG, Warn DE, Turner RM. Bayesian methods for analysis of binary outcome data in cluster randomized trials on the absolute risk scale. *Stat Med*. 2004;23(3):389-410. doi:10.1002/sim.1567
**M6**	Turner RM, Prevost AT, Thompson SG. Allowing for imprecision of the intracluster correlation coefficient in the design of cluster randomized trials. *Stat Med*. 2004;23(8):1195-1214. doi:10.1002/sim.1721
**M7**	Turner RM, Omar RZ, Thompson SG. Modelling multivariate outcomes in hierarchical data, with application to cluster randomised trials. *Biometrical J*. 2006;48(3):333-345. doi:10.1002/bimj.200310147
**M8**	Spiegelhalter DJ. Bayesian methods for cluster randomized trials with continuous responses. *Stat Med*. 2001;20(3):435-452. doi:10.1002/1097-0258(20010215)20:3<435::AID-SIM804>3.0.CO;2-E
**M9**	Kikuchi T, Gittins J. A behavioural Bayes approach for sample size determination in cluster randomized clinical trials. *J R Stat Soc Ser C Appl Stat*. 2010;59(5):875-888. doi:10.1111/j.1467-9876.2010.00732.x
**M10**	Turner RM, Thompson SG, Spiegelhalter DJ. Prior distributions for the intracluster correlation coefficient, based on multiple previous estimates, and their application in cluster randomized trials. *Clin Trials*. 2005;2(2):108-118. doi:10.1191/1740774505cn072oa
**M11**	Turner RM, Omar RZ, Thompson SG. Constructing intervals for the intracluster correlation coefficient using Bayesian modelling, and application in cluster randomized trials. *Stat Med*. 2006;25(9):1443-1456. doi:10.1002/sim.2304
**M12**	Uhlmann L, Jensen K, Kieser M. Bayesian network meta-analysis for cluster randomized trials with binary outcomes. *Res Synth Methods*. 2016;8(October 2015):236-250. doi:10.1002/jrsm.1210
**M13**	Turner RM, Omar RZ, Thompson SG. Bayesian methods of analysis for cluster randomized trials with binary outcome data. *Stat Med*. 2001;20(3):453-472. doi:10.1002/1097-0258(20010215)20:3<453::AID-SIM803>3.0.CO;2-L
**C1**	Peters TJ, Richards SH, Bankhead CR, Ades AE, Sterne JAC. Comparison of methods for analysing cluster randomized trials: An example involving a factorial design. *Int J Epidemiol*. 2003;32(5):840-846. doi:10.1093/ije/dyg228
**C2**	Pacheco GD, Hattendorf J, Colford JM, Mäusezahl D, Smith T. Performance of analytical methods for overdispersed counts in cluster randomized trials: Sample size, degree of clustering and imbalance. *Stat Med*. 2009;28(24):2989-3011. doi:10.1002/sim.3681
**C3**	Ma J, Thabane L, Kaczorowski J, et al. Comparison of Bayesian and classical methods in the analysis of cluster randomized controlled trials with a binary outcome: The community hypertension assessment trial (CHAT). *BMC Med Res Methodol*. 2009;9(1). doi:10.1186/1471-2288-9-37

Prefix “R” refers to results papers, “M” to methodological papers and “C” to comparison of methods papers

### Demographics

Descriptions of demographics are displayed in Table 3. Target sample sizes and numbers of clusters were only collected for primary results papers. We deemed it necessary to distinguish “numbers approached” from target sample sizes, as the numbers approached seemed likely driven by logistical rather than statistical considerations, and so were not included in the summary statistics of the target sample sizes. Clear statistician association with a CTU was identified in one (12.5%) study. We were unable to identify more general CTU involvement with trial or data management in any instance.

**Table 3:** Demographic data for the eleven results papers

***N*** (%) unless otherwise stated	Total (***N*** = 11)	Primary (***N*** = 7)	Secondary (***N*** = 4)
**Year of publication**			
Pre 2005	0 (0.0)	0 (0.0)	0 (0.0)
2005–2012	6 (54.5)	4 (57.1)	2 (50.0)
Post 2012	5 (45.5)	3 (42.9)	2 (50.0)
**Location of first author**^**a**^			
*UK*	2 (18.2)	1 (14.3)	1 (25.0)
*US/Canada*	5 (45.5)	4 (57.1)	1 (25.0)
*Europe excl. UK*	3 (27.3)	1 (14.3)	2 (50.0)
*Australia/New Zealand*	0 (0.0)	0 (0.0)	0 (0.0)
*Africa*	2 (18.2)	1 (14.3)	1 (25.0)
*Asia*	0 (0.0)	0 (0.0)	0 (0.0)
*Other*	0 (0.0)	0 (0.0)	0 (0.0)
**Location of study**^**a**^			
*UK*	1 (9.1)	0 (0.0)	1 (25.0)
*US/Canada*	3 (27.3)	3 (42.9)	0 (0.0)
*Europe excl. UK*	4 (36.4)	2 (28.6)	2 (50.0)
*Australia/New Zealand*	0 (0.0)	0 (0.0)	0 (0.0)
*Africa*	4 (36.4)	2 (28.6)	2 (50.0)
*Asia*	0 (0.0)	0 (0.0)	0 (0.0)
*Other*	0 (0.0)	0 (0.0)	0 (0.0)
Target sample size; mean (SD) [range]	N/A	*N* = 3^b^ 1466.7 (1868.6) [120, 3600]	N/A
Target number of clusters; mean (SD) [range]	N/A	*N* = 2^c^ 200.0 (198.0) [60, 340]	N/A
Recruited Sample Size; mean (SD) [range]	*N* = 11 10898.5 (19816.1) [116, 66204]	*N* = 7 2484.6 (3700.1) [116, 9928]	*N* = 4 25662.8 (28762.5) [683, 66204]
Recruited Number of Clusters; mean (SD) [range]	*N* = 11 58.8 (95.6) [5, 341]	*N* = 7 69.1 (121.6) [5, 341]	*N* = 4 40.8 (13.2) [21, 48]
**Randomisation unit**			
*Medical facility*	1 (9.1)	1 (14.3)	0 (0.0)
*Village/community/district*	6 (54.5)	4 (57.1)	2 (50.0)
*Organisation*	1 (9.1)	1 (14.3)	0 (0.0)
*Couple*	1 (9.1)	1 (14.3)	0 (0.0)
*Individual*	1 (9.1)	0 (0.0)	1 (25.0)
*Working unit (office)*	1 (9.1)	0 (0.0)	1 (25.0)
**Primary outcome type**			
*Binary*	9 (81.8)	5 (71.4)	4 (100.0)
*Continuous*	2 (18.2)	2 (28.6)	0 (0.0)
Statistician involvement	8 (72.7)	5 (71.4)	3 (75.0)
**Statistician association**			
*Clinical trials unit*	1 (12.5)	0 (0.0)	1 (33.3)
*Academic statistical department*	7 (87.5)	5 (100.0)	2 (66.6)
*Commercial pharmaceutical company*	0 (0.0)	0 (0.0)	0 (0.0)
*Clinical research organisation*	0 (0.0)	0 (0.0)	0 (0.0)
*Other*	0 (0.0)	0 (0.0)	0 (0.0)
**Journal endorsement of CONSORT guidelines**			
*High*	N/A	3 (42.9)	N/A
*Medium*	N/A	1 (14.3)	N/A
*Low*	N/A	0 (0.0)	N/A
*None*	N/A	3 (42.9)	N/A

^a^One author was associated with an institution in both Europe and the UK, and the associated study was run across both locations. The denominator used for the calculations is based on the number of papers

^b^Two studies specified the number of participants approached but these were not explicitly stated/justified recruitment targets and so were excluded

^c^Four studies specified the number of clusters approached but these were not explicitly stated/justified recruitment targets and so were excluded

### Reporting quality

Reporting quality of the seven primary results papers was mixed (Table 4). Four (57.1%) included a description of the sample size calculation, but none of these clearly accounted for clustering, provided the intra-class correlation coefficient (ICC) used in the sample size calculation or took into consideration potential variability in cluster size or accounted for this in the sample size calculation. Similarly, none of the papers reported estimated ICCs for any of the primary or secondary outcomes, despite the potential value of such estimates in informing the design of future studies. However, it was clear in six (85.7%) of the primary results papers how clustering was accounted for in the statistical analysis.

**Table 4:** Reporting quality metrics for seven primary results papers

Reporting quality criteria ***N*** (%)	Total (***N*** = 7)	Year of publication		Journal endorsement of CONSORT guidelines		Statistician involvement	
		2012 or earlier (*N* = 4)	2013 onwards (*N* = 3)	High/medium (*N* = 4)	Low/none (*N* = 3)	Yes (*N* = 5)	No (*N* = 2)
**Description of sample size method**	4 (57.1)	2 (50.0)	2 (66.7)	2 (50.0)	2 (66.7)	2 (40.0)	2 (100.0)
Was clustering clearly accounted for in sample size calculation	0 (0.0)	0 (0.0)	0 (0.0)	0 (0.0)	0 (0.0)	0 (0.0)	0 (0.0)
Specification of the required number of clusters	2 (50.0)	1 (50.0)	1 (50.0)	1 (50.0)	1 (50.0)	1 (50.0)	1 (50.0)
Specification of the assumed cluster size	2 (50.0)	1 (50.0)	1 (50.0)	1 (50.0)	1 (50.0)	1 (50.0)	1 (50.0)
Specification of whether equal or unequal cluster sizes are assumed	1 (25.0)	1 (50.0)	0 (0.0)	0 (0.0)	1 (50.0)	0 (0.0)	1 (50.0)
Variability in cluster size accounted for	0 (0.0)	0 (0.0)	0 (0.0)	0 (0.0)	0 (0.0)	0 (0.0)	0 (0.0)
Specification of the ICC used for the sample size	0 (0.0)	0 (0.0)	0 (0.0)	0 (0.0)	0 (0.0)	0 (0.0)	0 (0.0)
Indication of the uncertainty of the ICC	N/A	N/A	N/A	N/A	N/A	N/A	N/A
Accounted for the uncertainty in the ICC	N/A	N/A	N/A	N/A	N/A	N/A	N/A
**Other CONSORT metrics**							
Details of how clustering was accounted for in the analysis	6 (85.7)	4 (100.0)	2 (66.7)	4 (100.0)	2 (66.7)	5 (100.0)	1 (50.0)
Specification of the number of clusters randomised	7 (100.0)	4 (100.0)	3 (100.0)	4 (100.0)	3 (100.0)	5 (100.0)	2 (100.0)
Specification of the number of clusters receiving intended treatment							
*Explicit*	5 (71.4)	3 (75.0)	2 (66.7)	4 (100.0)	1 (33.3)	4 (80.0)	1 (50.0)
*Implied*	2 (28.6)	1 (25.0)	1 (33.3)	0 (0.0)	2 (66.7)	1 (20.0)	1 (50.0)
Specification of the number of clusters analysed for the primary outcome at the primary endpoint							
*Explicit*	2 (28.6)	1 (25.0)	1 (33.3)	2 (50.0)	0 (0.0)	2 (40.0)	0 (0.0)
*Implied*	5 (71.4)	3 (75.0)	2 (66.7)	2 (50.0)	3 (100.0)	3 (60.0)	2 (100.0)
Details of cluster-level losses and exclusions							
*Explicit*	3 (42.9)	2 (50.0)	1 (33.3)	2 (50.0)	1 (33.3)	2 (40.0)	1 (50.0)
*Implied*	4 (57.1)	2 (50.0)	2 (66.7)	2 (50.0)	2 (66.7)	3 (60.0)	1 (50.0)
Details of individual-level losses and exclusions	4 (57.1)	2 (50.0)	2 (66.7)	2 (50.0)	2 (66.7)	2 (40.0)	2 (100.0)
Individual-level baseline characteristics presented	7 (100.0)	4 (100.0)	3 (100.0)	4 (100.0)	3 (100.0)	5 (100.0)	2 (100.0)
Cluster-level baseline characteristics presented	2 (28.6)	2 (50.0)	0 (0.0)	1 (25.0)	1 (33.3)	1 (20.0)	1 (50.0)
**Coefficients of intracluster correlation provided for primary outcomes**							
*All*	0 (0.0)	0 (0.0)	0 (0.0)	0 (0.0)	0 (0.0)	0 (0.0)	0 (0.0)
*Some*	0 (0.0)	0 (0.0)	0 (0.0)	0 (0.0)	0 (0.0)	0 (0.0)	0 (0.0)
**Coefficients of intracluster correlation provided for secondary outcomes**							
*All*	0 (0.0)^a^	0 (0.0)	0 (0.0)^a^	0 (0.0)	0 (0.0)^a^	0 (0.0)	0 (0.0)^a^
*Some*	0 (0.0)^a^	0 (0.0)	0 (0.0)^a^	0 (0.0)	0 (0.0)^a^	0 (0.0)	0 (0.0)^a^
***P*-values provided for baseline comparisons**	5 (71.4)	3 (75.0)	2 (66.7)	3 (75.0)	2 (66.7)	3 (60.0)	2 (100.0)
**Clustering accounted for in the calculation of the** ***p*-values**							
*Yes*	1 (20.0)	1 (33.3)	0 (0.0)	1 (33.3)	0 (0.0)	1 (33.3)	0 (0.0)
*Unclear*	1 (20.0)	1 (33.3)	0 (0.0)	1 (33.3)	0 (0.0)	1 (33.3)	0 (0.0)

^a^One study did not have any secondary outcomes

Reporting quality metrics have also been summarised by the following: (i) publication date before or after the publication of the CONSORT extension to CRCTs in 2012 [12]; (ii) journal endorsement of the CONSORT guidelines [12]; and (iii) involvement of a statistician in the study (Table 4). Due to the small number of available papers, we dichotomised journal endorsement of the CONSORT guidelines into “High” or “Medium” versus “Low” or “None”. We intended to summarise these results by three time periods (pre-2005, 2005–2012 and 2012–2018) to assess any effect of the publication of the CONSORT extensions for CRCTs in 2004 and 2012 on reporting quality. However, we were unable to identify any CRCTs using Bayesian methodology published before 2005. Pre-specified quality metrics are detailed in Table 4. However, due to the small number of primary results papers identified (seven in total), no meaningful comparisons can be made.

One of the papers retrieved was a pre-specified sub-study and so was classified as a secondary results paper (Table 2, R10). We noted that reporting quality, despite not being a primary results paper and therefore not obligated to follow CONSORT guidelines, was high: a sample size calculation was presented and appropriately accounted for clustering, including specification of the assumed ICC; the flow of clusters and individuals through the study was well documented; and all levels of clustering were accounted for within a hierarchical modelling framework.

### Use of Bayesian methodology

We were unable to identify any results papers in which a Bayesian approach was taken, or even discussed, for study design or sample size calculation. One secondary paper did, however, specify that the design factor used to inflate the sample size calculation was derived from the results of a Bayesian hierarchical model.

Of the eleven results papers included in the review, all adopted some form of Bayesian approach to statistical analysis (Table 5). In nine (81.8%; R1–R7, R9, R10) of the 11 papers, hierarchical modelling techniques were employed to account for the clustered structure of the data. Another study employed Bayes Model Averaging (R8) in order to mitigate the risks of overfitting that can be associated with stepwise regression in model-fitting. One study conducted a literature search of Cochrane Reviews and extracted the key summary statistic (mortality) before converting each into a log-odds ratio. These statistics were combined into a single arithmetic mean in order to construct an empirical prior. This prior was then combined with the likelihood from the CRCT to obtain a Bayesian posterior distribution of the relative risk of mortality in the intervention group versus the control group (R11).

**Table 5:** Summary of Bayesian Methods used in primary and secondary results papers

***N*** (%)	Total (***N*** = 11)	Primary (***N*** = 7)	Secondary (***N*** = 4)
Sample Size (used)	0 (0.0)	0 (0.0)	0 (0.0)
Sample Size (discussed)	0 (0.0)	0 (0.0)	0 (0.0)
Analysis (used)	11 (100.0)	7 (100.0)	4 (100.0)
**Priors used**			
*Informative*	2 (18.2)^a^	1 (14.3)^a^	1 (25.0)
*Weakly Informative*	1 (9.1)	1 (14.3)	0 (0.0)
*Non-informative*	5 (45.5)^a^	3 (42.9)^a^	2 (50.0)
*Unspecified*	4 (36.4)	3 (42.9)	1 (25.0)
Analysis (discussed)	N/A	N/A	N/A

^a^One paper reported the use of two Bayesian models — the first model implementing a non-informative prior and the second model utilising “collateral” information

In these results papers, prior distributions were informative in two (18.2%; R3, R11) papers; in one, (R3) “collateral” information from a previous study was used to construct a prior distribution for the variation in practice effects (specifically, the standard deviation for practice-level rates); in the other (R11) an informative prior distribution for the treatment effect parameter within a negative binomial regression was constructed based on a meta-analysis of relevant reviews obtained from the Cochrane library, and used to inform the estimation of the outcome of interest (the relative risk of childhood mortality). No information was provided on the prior distributions placed on the variance components. Weakly informative prior distributions were used in one (9.1%; R2) study, by placing Student’s *t* priors centred at 0 on the treatment effect parameter and other fixed logistic regression coefficients, which the authors acknowledged would only affect inference if the data provide little information about the parameters. No detail was provided on the prior distributions specified for the variance components in this paper. Five (45.5%; R1, R3, R5, R9, R10) papers specified the use of non-informative prior distributions, although only one of these (R5) provided more specific detail, stating normal prior distributions for the treatment effect and each of the fixed logistic regression coefficients, and uniform prior distributions for the variance components. Four studies (36.4%; R4, R6, R7, R8) did not specify their choice of prior distribution. One paper fitted two Bayesian models (R3) - one model implementing a non-informative prior and the other utilising “collateral” information, so we recorded the use of both an informative and a non-informative prior.

### Bayesian methodological developments

We categorised 13 (48.1%) of the 27 papers included as methodological papers, where the focus was on the development of Bayesian methods for use in the design or analysis of CRCTs, as opposed to applying existing methods to data from CRCTs. Of these 13 papers, we defined 11 (84.6%) as “pure” methods papers, in which Bayesian methodological developments are reported independently of an applied scenario (although study data may have been used to demonstrate the method). We categorised two (15.4%) papers as being methodological but with the developments being driven by a specific statistical problem encountered in a CRCT, in which the method is presented and subsequently used to analyse the data of interest. Finally, we categorised three (11.1%) of the 27 papers as comparison of methods papers, in which existing methodology (both Bayesian and frequentist) were applied to the same data for comparative purposes.

Of the 11 “pure” methodological papers, seven presented analysis methods (63.6%; M2, M4, M5, M7, M11, M12, M13), two presented methods for design/sample size calculation (18.2%; M6, M9) and two presented elements of both (18.2%; M8, M10). Both papers driven by specific application presented analysis methods (M1, M3).

The analysis methods papers predominantly presented hierarchical modelling methodology applied to dealing with a range of data types, such as incidence rates (M1), count data (M2) and binary data (M4, M5,M13), in a Bayesian setting, citing flexibility of modelling and the ability to incorporate prior information and account for the complex variance structures as key advantages. One paper reports Bayesian methods for modelling multivariate outcomes (M7), which allow for multiple outcomes without concern for multiplicity whilst accommodating complex correlation structures. Another paper presents Bayesian network meta-analysis methods for CRCTs (M12), allowing for comparison of multiple treatment arms whilst accounting for the complex correlation structure inherent in clustered data.

A number of methodological papers identified within our review focus on the ICC. One such paper centres on analysis only, presenting methods for constructing intervals for the ICC and suggesting prior distributions for use in modelling (M11). The two papers in which both design and analysis are discussed focus heavily on the ICC; one provides a range of options for choice of prior distribution alongside recommendations, before discussing briefly how the uncertainty in the ICC can be accounted for in sample size calculations (M8). The other paper presents methods for formulating prior distributions for use in sample size calculations and statistical analysis on the basis of multiple previous estimates, whilst incorporating the relevance of the studies from which they were obtained (M10). One of the papers presenting only study design methodology also focused on ICCs, and developed methods to formulate prior distributions from single and multiple previous ICC estimates for use in sample size calculations (M6).

The remaining study design paper presented a behavioural Bayes approach (M9), extending existing methodology [26–29] for sample size determination in individually randomised trials to CRCTs. The method incorporates estimated financial costs and benefits of the intervention to produce a net benefit, rather than being based on the more usual difference in primary outcome alone.

## Discussion

To the best of our knowledge, this is the first methodological systematic review of the use, or consideration of, Bayesian methods in CRCTs.

As the number of included papers is small, drawing robust conclusions regarding overall reporting quality between subgroups (Table 4) is not possible. However, in 2013, Diaz-Ordaz presented a summary of reviews of CRCT quality, in which the percentage of studies accounting for clustering in the sample size calculation and statistical analysis ranged from 0% to 71% and 37% to 92%, respectively [15]. We have identified an additional review of reporting and methodological quality of CRCTs published in 2016 [16]. Including the data from the more recent review together with Diaz-Ordaz’s summary, the mean (SD) percentage of studies accounting for clustering in the sample size calculation and analysis was 34.6 (23.7) and 64.2 (16.3), respectively. For comparison, our study identified no papers which clearly accounted for clustering in the sample size calculation, and six (85.7%) papers accounting for clustering in the analysis. Although our review included only a small number of papers, reporting quality according to these key metrics may differ somewhat between studies using Bayesian methodology and the wider pool of CRCTs, as none of the papers we identified clearly accounted for clustering in sample size calculation. Hence, there is a need to further improve the reporting of CRCTs utilising Bayesian methodology. Conversely, Bayesian CRCTs seem to more often account for clustering in analysis. This is likely due to the popularity of Bayesian hierarchical modelling within the set of included papers, which is a natural way to conduct mixed or random effects modelling and therefore inherently account for clustering.

Evidently, the use of Bayesian methods in the design or analysis of CRCTs remains uncommon relative to the use of frequentist methods (Fig. 1), with only eleven primary or secondary results papers reporting doing so. This is despite the increasing use of CRCT designs, with over 120 reported in 2008 alone [4] and the number of PubMed search results rising almost year-on-year since 2006 (Fig. 1) reaching 347 in 2018. This methodological systematic review failed to identify a single reported CRCT which utilised a Bayesian approach to conduct the sample size calculation, despite some efforts to develop methodology in this area, as highlighted in the methodological aspect of our review. Whilst explaining the reason for this lack of uptake of Bayesian methodology in the design of CRCTs would be little more than speculation, possibilities include fundamental disagreements with the approach, still limited development of methodology, inaccessibility of software to implement the methods or lack of knowledge or understanding. Whilst we have shown that there has been some Bayesian methodological developments in both design and analysis of CRCTs, these have been limited in comparison to the development of classical methods which are now well-established in the literature. None of the thirteen published methodological papers appears to have developed publicly available software in order to aid implementation (although some papers reported that code is available from the authors on request), whereas classical analysis and sample size calculations for CRCTs can be conducted with relative ease in standard statistical software. As such, there is need to increase the availability and accessibility of these methods, which can offer advantages over the frequentist approach within the CRCT context.

A common criticism of the Bayesian approach in general, and in particular within the analysis of clinical trial data, is the subjective nature of the choice of prior distribution, although it is strongly recommended that sensitivity analyses be performed in order to assess the strength of the effect of the prior [30]. Interestingly, however, only two (18.2%) of the 11 results papers that were identified utilised an informative prior distribution, and one (9.1%) utilised a weakly informative prior. Five (45.5%) specified an uninformative prior (of which one employed two models). It is likely that the four (36.4%) papers that did not report their choice of prior used an uncontroversial, uninformative formulation, and in doing so, a likely total of nine (81.8%) studies circumvented the perceived issues surrounding the choice of an informative prior. Despite this, the use of a well-justified, informative prior distribution has the potential to add value to a statistical analysis, and methodological development for informative yet rigorous prior specification for CRCTs may enhance the uptake of Bayesian methods in this area.

### Strengths and limitations

A protocol for this methodological systematic review was published before commencement of the electronic search [17] and the review was conducted according to the PRISMA guidelines [18]. The electronic search strategy to identify Bayesian approaches in CRCTs was adapted from a previously published strategy, which was demonstrated to have high precision [20] in identifying CRCTs. In this study, each stage of the reference sifting and data extraction process was fully conducted twice, independently, to ensure accurate inclusion of references and high-quality data for examination. We developed data extraction forms for primary and secondary results papers in order to aid in the accurate and consistent collection of data. Furthermore, the final data extraction was agreed by all four members of the study team.

The reporting quality metrics collected are predominantly a subset of the CONSORT checklist for CRCTs, a well-accepted set of criteria. We added a small number of additional items such as whether cluster size variability had been accounted for in the presented sample size calculation [4] and whether *p*-values for baseline comparisons were provided, in order to facilitate a robust judgement of reporting quality.

Despite this, we acknowledge the possibility that we may have missed some publications in which Bayesian methodology was used or considered in the design or analysis of CRCTs. In particular, we opted for a search strategy in which specificity was maximised, rather than sensitivity, in order to make the sifting process more manageable with limited resource. We added six additional methodological papers through hand searching, but were unable to identify any additional trial results papers. This is not surprising given the search strategy was developed to identify the latter, but may suggest a greater risk that further methodological papers have been missed compared to trial results papers.

Furthermore, we present reporting quality metrics by journal endorsement of the CONSORT guidelines. However, we acknowledge that the guidelines may, in some cases, have changed since the date of the associated publications, and as a result, a journal’s endorsement may have been intensified since the included papers were accepted for publication. To the best of our knowledge, this issue has not been raised in previous systematic reviews of trial reporting quality; archiving of journal guidelines would help researchers conducting quality assessment systematic reviews in the future. Similarly, we sought to identify author affiliations during data collection, but again acknowledge that these may have changed since publication of the research, particularly for papers published some time ago.

We intended to summarise the pre-specified reporting quality metrics by time periods (pre-2005, 2005–2012 and 2012–2018) according to publication date to assess the effect of the relevant CONSORT statements on reporting quality. We acknowledge that the time delay between completion of the study and submission of the final report for publication may have resulted in some studies being categorised as published after the publication of the CONSORT extension guidance, when in fact it was designed, conducted and possibly even analysed before.

## Conclusion

The use of Bayesian methods in the statistical analysis of CRCTs is rare and was not found at all in the design of any of the reviewed studies or their sample size calculations. There have been some developments in Bayesian methodology for CRCTs but far less so than within the frequentist paradigm. Reporting quality may differ between CRCTs utilising Bayesian methodology compared with previous reviews of CRCT quality, although the number of papers identified in this review is small. There is a need for further Bayesian methodological developments in the design and analysis of CRCTs, including approaches for the specification of prior distributions, as well as statistical software development to allow easier implementation of methods, in order to increase the accessibility, availability and, ultimately, use of the approach.

## Data Availability

The datasets generated and/or analysed during the study are available on request.

## Abbreviations

CENTRAL: Cochrane Central Register of Controlled Trials
CRCT: Cluster randomised controlled trial
CRO: Clinical research organisation
CTU: Clinical trials unit
ICC: Intra-class correlation coefficient
RCT: Randomised controlled trial
